# Gynecological cancers: an alternative approach to healing

**DOI:** 10.4155/fsoa-2017-0022

**Published:** 2017-07-12

**Authors:** Srdjan Saso, Benjamin P Jones, Timothy Bracewell-Milnes, Gulsen Huseyin, Deborah C Boyle, Giuseppe Del Priore, James Richard Smith

**Affiliations:** 1 Division of Surgery & Cancer, Institute of Reproductive & Developmental Biology, Imperial College London, Hammersmith Hospital Campus, Du Cane Road, London W12 0NN, UK; 2 North Paddington Community Mental Health Service, 7a Woodfield Road, London W9 2NW, UK; 3 Royal Free Hospital, Pond Street, Barnet NW3 2PN, UK; 4 Department of Gynaecologic Oncology, Indiana University, IN 46202, USA; 5 West London Gynaecological Cancer Centre, Queen Charlotte's & Chelsea Hospital, Hammersmith Hospital Campus, Imperial College London, Du Cane Road, London W12 0NN, UK

**Keywords:** cancer, coping strategies, doctor–patient communication, gynecology, holistic approach, psychology, religion, spirituality

## Abstract

Grief and hope are two conflicting emotions that a patient recently diagnosed with cancer has to master. The real challenge for gynecologic oncologists is how to reach out. Conventional wisdom states that offering patients focus and belief when combating cancer in their lives allows them to embrace hope with greater confidence, which minimizes their grief. Three pictorial models are presented: ‘4-cusp approach’ model used at the initial consultation; ‘tapestry of bereavement or landscape of grief’ model at the postsurgery consultation; and ‘Venn-diagram’ model at any time during patient management. We have applied these models in our practice and believe that they can act as a fulcrum for the patient, the family and healthcare team around which therapy should be centered.

## Lay abstract

Grief and hope are two emotions that a patient faces if diagnosed with cancer. The real challenge for the doctor is how to reach out and help the patient through this process. A doctor's role may be to offer focus and belief to the patient which may allow her to embrace hope with greater confidence. This will hopefully lessen the grief. We present three models which we believe can play a crucial part: ‘4-cusp approach’ used at the initial consultation; ‘tapestry of bereavement or landscape of grief model’ at the postsurgery consultation; and ‘Venn-diagram model’ at any time during care.

Communicating with patients is a skill that doctors have traditionally found very challenging, both at junior and consultant levels. This particular challenge grows in terms of scale when the added element of a life-threatening diagnosis is discussed. Contrary to what many people believe, the worst part of a cancer journey is not the day a patient is told that he or she has cancer or even the period when they undergo treatment. The hardest period is often when the treatment has finished and they are told to go and live life almost as if nothing has happened. Patients, like everyone else, tend to either have a ‘cup half-full’ optimistic attitude to life, or the opposite, ‘cup half-empty’ approach. Generally speaking, the former tend to live well with their diagnosis, fighting it head on, whereas the latter spend the time following their treatment frightened of possible recurrence. The real challenge for doctors and oncologists in particular is how to reach out to this latter group and by doing so, alter their viewpoint of their cancer diagnosis and lifestyle in general.

Nowadays, many cancers have excellent prognoses. In gynecology, for example, most patients diagnosed with cancer will be cured, with a great majority living past the 5-year survival mark [1]. With respect to gynecological cancer, a patient is considered to be cured if she has remained disease-free for 5 years following treatment. At that point, a patient is no longer a patient in the medical sense of the world and should live as long as any person that never had the disease.

Such favorable outcomes contrast greatly with the situation 20 years ago and, therefore, the ability of a patient to live positively with their diagnosis has become much more important. It is our duty as doctors to try to make such a scenario a reality, by exploring with them the mechanisms that they can use to cope better with their disease.

Our team practices three different pictorial models [2]:

- Initial consultation: the 4-cusp approach model;
- Postsurgery consultation: tapestry of bereavement or a landscape of grief model;
- General approach to living with cancer: Venn-diagram model.

We describe them here because we believe passionately in patient-centered care. Our sole aim is not to impose our way of thinking, but to share our experiences of trying to turn the ‘cup half-empties’ into ‘cup half-fulls’.

## Dealing with cancer: the initial steps

*“…In my Lucia's absence Life hangs upon me, and becomes a burden; I am ten times undone, while hope, and fear, And grief, and rage and love rise up at once, And with variety of pain distract me…”*

Joseph Addison (1 May 1672–17 June 1719), English essayist, poet, playwright and politician.

As we have stepped into the 21st century, the approach to treating cancer has changed. Orthodox medical treatments provide the mainstay of treatment, with a concentrated effort to improve them using scientific rigor. However, we have also witnessed a steady increase in the use of a plethora of complementary therapies by cancer patients, ranging from acupuncture to homeopathy, hypnotherapy, meditation, spiritual healing and reiki [3,4]. The medical profession, although skeptical at first, has slowly begun to accept the alternative types of therapeutic methodology, provided it leads to a noticeable benefit to the patient. Each patient is now much more likely to be treated on an individual basis. If a particular therapy allows a patient to cope with their diagnosis, treatment and follow-up with greater confidence and hope, then, it should be added to the ‘anticancer artillery’.

Coping with cancer is a multifaceted concept that patients try to master after their initial diagnosis. Central to it is the relationship between the patient and the care staff (particularly, cancer nurse specialists and doctors), acting as a springboard for all other interactions (Box 1). A positive approach of the patient to her cancer should begin with the initial consultation. The doctor has a unique chance to form a trusting bond and offer support, thus allowing the patient to turn a situation filled with sadness and anxiety into one where she can face the future with optimism and energy. The patient can also face the future with the knowledge that she is not alone during her battle to beat the disease.

A particular approach, utilizing a combination of the aforementioned three pictorial models, has been developed and employed by this set of authors over many years of gynecological oncology consultations. It has been utilized when trying to explain to patients their diagnosis of cancer, through the lenses of grief and hope. Initial reports from the patients and their families have been encouraging, with the approach seemingly helpful to many women over the years. It has also allowed them to explain to their families the situation they find themselves in and where they are in the cancer management process. We believe that it has the potential to disperse the ‘clouds of grief and demonstrate the powerful elixir of hope’. As it was developed for gynecological patients initially, we will treat our patients as ‘female’ throughout the article.

## The concept of hope

*“For in hope we have been saved, but hope that is seen is not hope; for why does one also hope for what he sees? But if we hope for what we do not see, with perseverance we wait eagerly for it.”*

Romans 8: 24–25.

The importance of hope is usually first conveyed to the patient by her family and friends. Likewise, it is the doctor's responsibility to strongly convey the message of hope to the patient and her family. This is of vital importance to setting the patient on the appropriate path to deal with the diagnosis. Hope plays a big role to three groups: the patient, the patient's family and the medical team. The majority of patients must have hope that they will be cured. For those who do not get cured, there is real hope of a number of years of good quality living. Patients who endure disease progression can have hope of excellent symptom control and comfort. Finally, for those patients who have sadly reached the end of their time, there is hope for peace and dignity and death without pain.

The passage from the Bible above defines hope as a spiritual grace that must be attained to allow us to overcome particular issues. It is a belief in a positive outcome of an event in one's life. Hope involves the expectancy of positive outcomes, and in particular, the ability to see how those positive outcomes can be reached. The ancient Greeks had Pandora, the first woman on Earth, to thank for closing her box just before hope could also escape along with all the evils of the world. Thus, we were left with hope to help us with evils that might accost us in the future.

## The 4-cusp approach to cancer care

*“What are days for?*

*Days are where we live.*

*They come, they wake us*

*Time and time over.*

*They are to be happy in:*

*Where can we live but days?*

*Ah, solving that question*

*Brings the priest and the doctor*

*In their long coats*

*Running over the fields.”*

Philip Larkin (9 August 1922–2 December 1985), ‘*Days*’.

The concept of the ‘4-cusp approach’ is to allow the patient to move from the positions of despair and anger to one of hope, thus allowing active involvement in her own management [1]. It acts as a map for all cancer patients and is a very useful method of communicating concepts of cure, as well as living and dying with cancer. It is not a clinical model and bears no relationship to the four International Federation of Gynaecology and Obstetrics (FIGO) pathological stages of cancer (I, II, III and IV). This approach to patient care has been developed over many years, fueled by a vast number of interactions with patients in clinics and on the wards. What was evident very early on was a need to map out clearly a pathway for the patient to follow and to rely on once the diagnosis of cancer had been made. This pathway is based on three important factors (physical, psychological and life expectancy/quality) whose aim is to depict the patient's position in relation to the cancer.

The ‘4-cusp approach’ is an interactive process applied at the first consultation involving the patient, patient's family if present and the gynecological oncologist. The patient is actively involved in dealing with the above three factors. Furthermore, the question, ‘how long have I got’ (which should never be answered directly by a doctor), is often asked, and it may be tempting to hazard a guess but this can so often be very inaccurate as to cause false hope or misery, so we caution very strongly against answering this question directly even if pressed.

**Figure 1.**
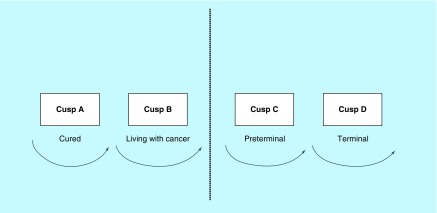
**The ‘4-cusp’ approach showing figure a row of four cusps.** Each cusp represents a patients’ state in terms of cure and life expectancy. Reproduced with permission from [2].

It can be dealt with using a variance of our approach. The ‘4-cusp approach’ is represented pictorially (Figure 1), and the ‘map’ to having cancer has seemed to resonate with patients and their families. This team has spent considerable time drawing pictures of surgical operations that patients were to have. Infrequently has the picture been taken for future reference. However, the 4-cusp diagram has been taken away by numerous patients who have found it very helpful, both for themselves and to assist in further dialog with their families. Indeed, symbols can be hugely beneficial when dealing with emotive concepts, such as recent cancer diagnosis, that are usually difficult to explain with language.

We can see from the figure a row of four cusps. Each cusp represents a patients’ state in terms of cure and life expectancy (Table 1). The two most important aspects of the model are the action of folding down the central dotted line and the ‘circle’ aimed at in both cusps A and B. This ability to fold the paper and thus place cusps C and D out of sight, coupled with the powerful image of a circle which in a Judeo-Christian culture suggests ‘holism’. This appears to give a patient a psychological strength at the beginning of what is going to be a highly arduous journey. They are, in our opinion, the model's strongest features. It is noteworthy that patients have produced the 4-cusp drawings from their wallets many years after the primary diagnosis and it always folded!

**Table 1.** **Cusps representing a patients’ state in terms of cure and life expectancy.**

**Cusp**	**Cure potential**	**Life expectancy**
A	Cured	Months to years

B	Living with cancer	Months to years

C	Preterminal	Weeks to months

D	Terminal	Hours to days

Cusp definitions reproduced with permission from [2].

### First consultation

When the diagnosis of cancer is conveyed to a patient for the first time in a consultation, she experiences a frightening and stressful event. Understandably, the rest of the consultation is almost always forgotten, with only the actual diagnosis remembered. By using the 4-cusp diagram, the patients are given an object that they can focus on. We then proceed and explain that the vast majority of our patients are not in cusps C and D, but either in cusp A or B. This allows us to fold the paper down the dotted line and literally put cusps C and D out of the way. This action of removing the two cusps most associated with death provides the patient with the aforementioned psychological boost, at a time when she needs it the most [2].

Emphasis should be placed on giving patients as much hope as is appropriate. The golden rule is to impart the truth in as gentle fashion as possible. It should be stressed that just as all patients are different, so are all cancers. We tend to point out that the majority of gynecological tumors are cured in the long term, and try to disperse the ‘death sentence’ type of atmosphere. The outlook should remain positive, with special importance placed on forming a ‘dynamic’ doctor–patient relationship.

The remainder of the consultation should be spent on developing an outline of a timetable as to when investigations will be done, and when and where surgery or treatment will take place. The patient must be notified of a specific date when full results, including exactly how far the cancer has spread, will be provided. On that date, it will be possible to talk far more accurately about the patient's outlook and prospects. The specific timetable to confirm the diagnosis, undertake any surgery or biopsy and obtain the results of the analysis of the removed cancer should take 2–4 weeks approximately.

### The cusps explained

**Figure 2.**
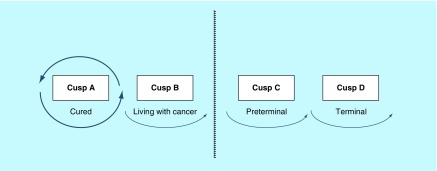
**Cusp A: the cured circle.** If it is demonstrated that the cancer has been completely removed, the conclusion is that the patient is presumed cured, in other words, she remains at cusp A. Reproduced with permission from [2].

**Figure 3.**
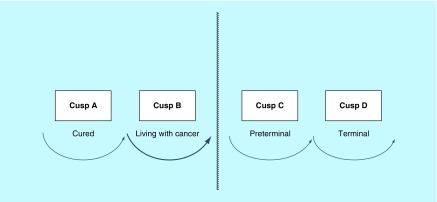
**Cusp B: diagnosis.** Following adjuvant treatment with chemo- or radiotherapy, the patient may or may not be cured. She is psychologically ‘living with her cancer’ and should recognize that at this point she is not dying from her disease. Reproduced with permission from [2].

Cusp A or B applies to the patient from the time of the first visit to the clinic, when the clinician explains the likely diagnosis and discusses further management with respect to how advanced the cancer is. Following surgery or biopsy and consequent histopathology results, the oncology team will have a better idea of future prognosis. Two options are possible. If it is demonstrated that the cancer has been completely removed and there is no distant spread, the conclusion is that the patient is presumed cured, in other words, she remains at cusp A. If she is in remission 5 years after surgery, the word ‘presumed’ can finally be omitted – the patient is indeed cured at that point in time (Figure 2). However, if during surgery, it is shown that the tumor has spread to the lymph nodes or beyond, the assumption is that the patient has entered cusp B (Figure 3). Following adjuvant treatment with chemo- or radiotherapy, she may or may not be cured. She is psychologically ‘living with her cancer’ and should recognize that at this point she is not dying from her disease [2].

**Figure 4.**
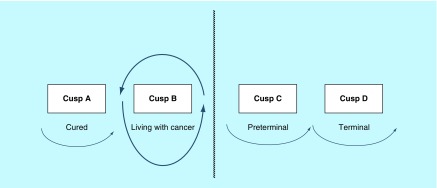
**Living with cancer circle.** A good response is found initially but the patient develops recurrence of her cancer. She is treated again with the same adjuvant therapy and remains in cusp B. Reproduced with permission from [2].

**Figure 5.**
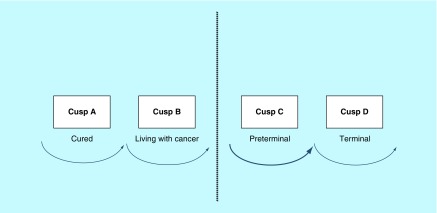
**The ‘preterminal cusp’.** A poor response is observed however following treatment of the recurrence – this patient has now entered cusp C. Reproduced with permission from [2].

The likelihood is that the patient who is within cusp B will need further therapy, either in the form of chemotherapy or radiotherapy. The response to adjuvant therapy will determine the patient's path. Three scenarios are possible:

- Excellent response and at 5 years, the patient has no sign of tumor recurrence; thus, she has moved into the ‘cured’ circle A;
- A good response is found and the patient remains well for 4 years, at which point she develops recurrence of her cancer. She is treated again with the same adjuvant therapy as before with good prospects of a similar 2–4-year response. Thus she remains in cusp B (Figure 4);
- A poor response is observed, with the cancer recurring 5 months later. This patient has now entered cusp C (Figure 5).

The patient who has entered cusp C will need the most support and time. She has very little chance of cure and has entered the preterminal phase of her illness. These issues are discussed with the patient and her family. Interestingly, many patients have said to the authors at this type of follow-up consultation ‘have I just moved to cusp C?’, making this most difficult of discussions slightly easier.

Her treatment options are now more palliative in nature, in other words, the maximum quality rather than quantity of life. Additionally, the patient might have a spiritual need that needs exploring, in particular, a search for meaning. Patients begin to feel a sudden need to repent, be valued, achieve self-fulfillment and, in the process, face and accept death [5]. They are looking for a spiritual resolution, in other words, verification and appreciation of relationships with one's self, family, friends, community and, in some cases, a religious deity [5,6].

### Evaluation of the 4-cusp approach

Huseyin explored the impact of doctor–patient communication on the psychological adjustment of a patient to gynecological cancer [Huseyin G, unpublished data]. Patients were interviewed about their experiences across all stages of professional care, from diagnosis to recovery. The interpretative phenomenological analysis method was applied to analyze interview transcripts. The themes identified in this qualitative study describe women's susceptibility to feelings of vulnerability and loss of control over the illness and their bodies. The findings demonstrate that communication with health professionals is likely to play an important role in promoting feelings of control, particularly during treatment planning.

Use of the 4-cusp approach, for example, was found to be useful in enabling women to manage their uncertainty and maintain hope (Supplementary Material A). Twenty-two percent of the women interviewed spoke of how communication strategies like the 4-cusp approach had enabled them to feel in control and hopeful about the future. Written information presented diagrammatically had helped them to clarify, remember and hold onto the hopefulness and certainty about the healthy futures their doctors anticipated for them. They described how this approach provided them with clear and simple prognostic information at both the diagnostic and follow-up stages of their care. The diagram had helped these women to delineate themselves from those who were dying from cancer.

The 4-cusp approach may offer an alternative means of conveying hope to statistical information about cancer recurrence rates. Women may understand concepts of cure, living and dying with cancer more concretely, by allowing them to see where they are with the disease and refuting thoughts of dying on hearing they have cancer. Butow *et al*. found that women with breast cancer sought hope and reassurance from professionals, as opposed to life expectancy statistics [7]. Straightforward, factual information about prognosis also enables patients to arrange their coping resources around a ‘realistic and predictable future’. The 4-cusp approach may therefore promote feelings of hope in this way.

## Bereavement & grief

*“…The stages (of the model) have evolved since their introduction, and they have been very misunderstood over the past three decades. They were never meant to tuck messy emotions into neat packages. They are responses to loss that many people have, but there is not a typical response to loss. There is no typical loss. Our grief is as individual as our lives…”*

Elisabeth Kübler-Ross (8 July 1926–24 August 2004), from ‘*On Grief and Grieving: Finding the Meaning of Grief Through the 5 Stages of Loss*’.

Bereavement is, by definition and also the above description, an individual process that a person goes through when he or she suffers a loss of something or someone to which a connection was formed. A negative reaction to that loss is defined as grief. Both bereavement and grief are multidimensional concepts; they deal with emotional, physical, social and philosophical ideas. With respect to our patients, the terms apply to women diagnosed with cancer who are coping with the loss that this entails and to a lesser degree to their relatives at the time. They clearly apply very strongly to the relatives of the minority of women who sadly succumb to the disease, creating an effect similar to ‘ripples of water after throwing a stone into a still lake’.

Traditionally, bereavement has been seen in a stepwise progression, as described by the Kübler-Ross model, first introduced by Elisabeth Kübler-Ross in her 1969 book, “On Death and Dying” [8,9]. Patients move through five discrete stages, denial, anger, bargaining, depression and acceptance/hope, with a new sense of being [8,9]. Therefore, life will never be the same again, but it can go on, albeit differently. The five stages form a framework which identifies clearly how a patient might be feeling. It was designed to guide and not a strict, prescribed order.

**Figure 6.**
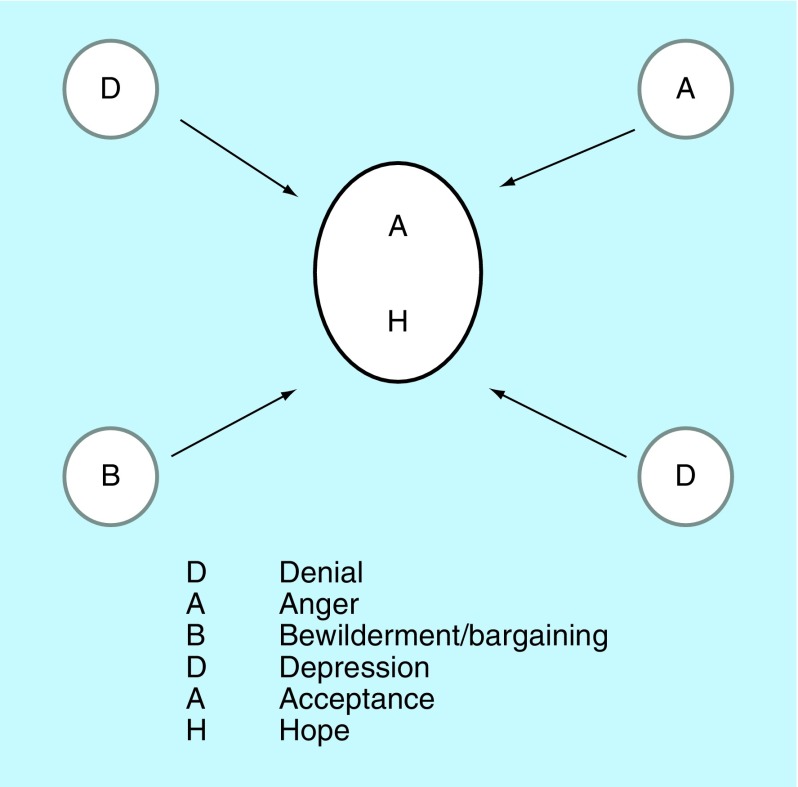
**‘Tapestry of bereavement or a landscape of grief’: the DABDAH model.** The various emotions crop up not in any particular order but more randomly, with some predominating at one point and others at another. Over time, acceptance and hope arrive (represented by the letters A and H at the center of the figure), causing other parts of the tapestry to fade. Reproduced with permission from [2].

A different model encompassing the same emotions is the ‘tapestry of bereavement or a landscape of grief’ model [2]. It is applied at the first consultation after surgery. At this point, we are able to inform the patient of the histology results, which in most cases, are positive for cancer. The analogy for this model is one of a painting. When one buys a painting, takes it home and hangs it on the wall, various features of it are noticed, but over time, different aspects of the painting appear in view. Finally, after some time, the painting is hardly noticed at all, even though it is still there. Figure 6 illustrates our painting analogy. The various emotions crop up not in any particular order but more randomly, with some predominating at one point and others at another. Over time, acceptance and hope arrive (represented by the letters A and H at the center of the figure), causing other parts of the tapestry to fade. Even though they do not disappear, the tapestry becomes the patient's new reality.

## Psychology, religion & spirituality: an obvious overlap

*“.From where the Western seas gnaw at the coast of Iona.*

*To the death in the desert, the prayer in forgotten places by the imperial column.*

*From such ground springs that which forever renews the earth though it is forever denied”*

T S Eliot (26 September 1888–4 January 1965)

from ‘*Murder in the Cathedral*’.

For us to discuss these topics brings on a number of varied responses, ranging from ‘brave’, ‘foolhardy’, to ‘presumptuous’ and even ‘inappropriate’. The three terms (psychology, religion and spirituality) encompass a broad number of topics which are beyond the scope of this work. By highlighting them here, our aim is to point out that from our shared experiences, religion, spirituality and psychology have allowed our patients to cope better with their disease. Spirituality is used to describe the attempt to experience a sense of the transcendental, independent of religion.

**Figure 7.**
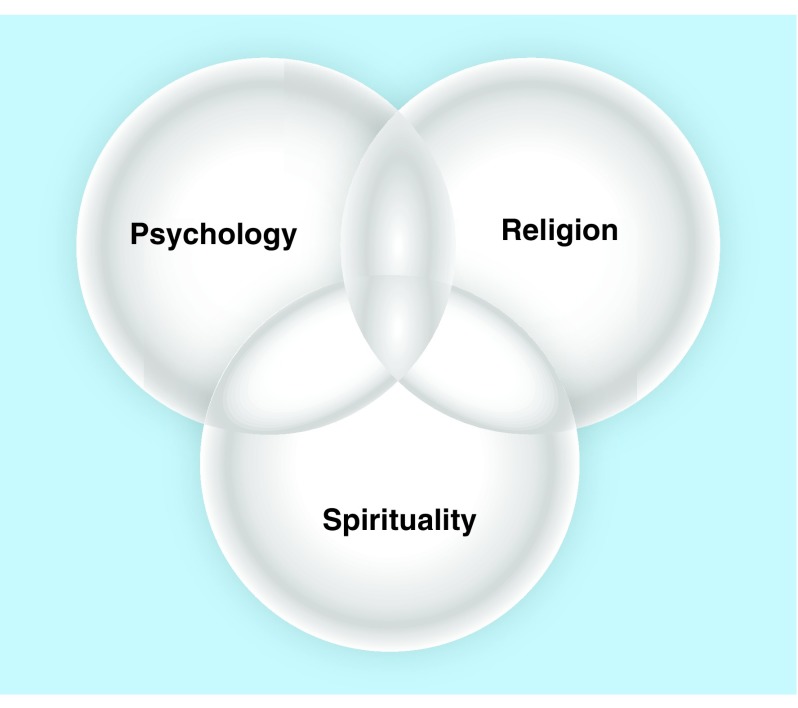
**Venn diagram: crossover between sprituality, psychology and religion.** When it comes to living with cancer, they all seem to have the capacity to provide reassurance and hope in equal measure to each other. Reproduced with permission from [2].

There is a common thread running through all of the world's main religions, the concept of spirituality, as well as psychology. The Venn diagram in Figure 7 illustrates this crossover, while simultaneously highlighting their uniqueness [2]. When it comes to living with cancer, they all seem to have the capacity to provide reassurance and hope in equal measure to each other. It is noteworthy that hospitals historically, certainly in Western tradition, grew out of monasteries where a belief existed in tending to the physical, emotional and religious well-being of those being cared for. Our thought is that this points to a particular failing of modern medicine, in other words, as we have got better at achieving higher cure rates, and have much more to offer as each year goes by, we have left the care of the spiritual side of the patient behind almost as a matter of irrelevance, to the point of denying its existence. Worse than this, it is almost impossible to enter the arena of spirituality and religion with your work colleagues or patients without a serious danger of being seen as too ‘alternative’, not scientifically rigorous enough, or even worse, proselytizing for one's own particular brand of religion. In worst case scenarios, such action could even result in the doctor or nurse becoming unemployed. In 2009, rather shockingly a nurse was suspended for simply offering to pray for an elderly patient's recovery.

The great difficulty lies in how to initially approach the subject with a particular patient. To us, it is only sometimes possible, and almost always when we have truly managed to get to know a patient on a more personal level. However, by drawing the Venn diagram and saying, ‘do any of these areas apply to you?’, one can enter this arena in a nondirective fashion.

## Future perspective

*“…Of splendour in the grass, of glory in the flower…”*

William Wordsworth (7 April 1770–23 April 1850), from ‘*Ode, Intimations of Immortality from Recollections of Early Childhood*’.

A qualitative study looking more closely at the above approaches would be welcome. Equally, we feel that an overlap which clearly exists between the areas of psychology, religion and spirituality is a subject that many patients hope or expect their doctors to touch upon and discuss with them. It is our responsibility not to shirk away from this challenge but to, instead, find appropriate and culturally acceptable ways of broaching such topics with our patients.

**Box 1.**  **Golden rules of cancer management.**

- Always impart the truth, in as gentle fashion as possible. Never tell lies
- Never say never. If the patient and the doctor believe that they are beaten before the start, then they are!
- As doctors, we should never say there is nothing more that we can do for the patient – there always is!
- The patient and the doctor are on the same team and should be working toward the same commonly understood and shared goals: quantity of life, but only if accompanied by quality
- Spiritual peace does not necessarily increase longevity but those patients with it seem to fare better in the cancer process than those without it. All patients should consider exploring this, with the understanding that a number of pathways do not involve any religion

Reproduced with permission from [2].

**Executive summary**

**Background**

- Grief and hope are two conflicting emotions that a patient has to master.
- Conventional wisdom states that offering patients focus and belief when combating cancer in their lives allows them to embrace hope with greater confidence.
- We have successfully applied the ‘4-cusp approach’, ‘tapestry of bereavement or a landscape of grief’ (the DABDAH model) and ‘Venn diagram’ models in our practice.
- We believe that they can act as a fulcrum for the patient, the family and healthcare team around which therapy should be centered.

**Dealing with cancer: the initial steps**

- Coping with cancer is a multifaceted concept.
- Central to it is the relationship between the patient and the care staff acting as a springboard for all other interactions.
- A positive approach to cancer should begin with the initial consultation.

**The concept of hope**

- The importance of hope is usually first conveyed to the patient by her family and friends.
- Likewise, it is the doctor's responsibility to strongly convey the message of hope.
- This is of vital importance to setting the patient on the appropriate path to deal with the diagnosis.

**The 4-cusp approach to cancer care**

- The ‘4-cusp approach’ acts as a map for all cancer patients and is a very useful method of communicating concepts of cure, as well as living and dying with cancer.
- It is not a clinical model and bears no relationship to the four FIGO pathological stages of cancer (I, II, III and IV).
- This pathway is based on three important factors (physical, psychological and life expectancy/quality) whose aim is to depict the patient's position in relation to the cancer.
- The ‘4-cusp approach’ is an interactive process applied at the first consultation involving the patient, patient's family, if present and the gynecological oncologist.

**Bereavement & grief**

- Both bereavement and grief are multidimensional concepts.
- They deal with emotional, physical, social and philosophical ideas.
- The terms apply to women diagnosed with cancer who are coping with the loss that this entails and to a lesser degree to their relatives at the time.

**Psychology, religion & spirituality: an obvious overlap**

- Religion, spirituality and psychology have allowed patients to cope better with their disease.
- When it comes to living with cancer they all seem to have the capacity to provide reassurance and hope in equal measure to each other.

**Future perspective**

- A qualitative study looking more closely at the above approaches would be welcome.
