# Supplementary material for: Gynecological cancers: an alternative approach to healing
Source: Future Sci OA. 2017 Jul 12;3(3):FSO208. doi: 10.4155/fsoa-2017-0022 (PMC5583663; doi:10.4155/fsoa-2017-0022)
Supplement: Supplementary file 1 [file fsoa-03-208-s1.docx]

**Supplement A**

**Excerpt taken from - Super-ordinate theme: The invisible illness: managing feelings of uncertainty about cancer recurrence** [10]

**Sub-ordinate theme: Maintaining hope**

Melissa and Deidre^[[1]](#endnote-1)^ explained how written and diagrammatic information provided by the consultant surgeon had helped them to maintain hope, despite the uncertainty they may have felt about their future health status:

“…he [consultant surgeon] did this little diagram [4-cusp model] of where I am and where people with cancer could be on it…it was just a simple way of communicating…something well. It really - I realised my God I’m fine…Just through a little bit of paper and a pen and that was excellent, that sticks in the mind…” (Melissa; 1045)

“… because it’s stuck in my mind, the fact that I was at the very distant - first part of this diagram whereas the people at the end were dead. You know! But I was right here and I didn’t need to take it away with me really. I just thought fine I know where I am.”

(Melissa; 1126)

Having prognostic information presented in this way enabled Melissa to create a mental boundary between illness and recovery:

“ …if I wanted to look at it I had it…If I just wanted to remind myself look where we are now… it really, really helped…If he hadn’t done a little diagram I would have got the same impression but it was a good simple means of communicating the facts, and that was good, I thought yep that’s fine.” (Melissa; 1137)

Both Melissa and Deidre describe how having this clearly written diagram enabled them to feel hopeful and more certain about the future outside of follow-up appointments with their consultant. They found it helped them to manage their feelings and concerns about their health status:

“It made it seem a little less scary I think…” (Melissa; 1161)

“It’s quite a helpful way of…dealing with it.” (Deidre; 239)

Deidre was the only participant who had been diagnosed and treated for a gynaecological cancer before. She described how the Consultant Surgeon’s ongoing use of the diagram had allayed her fears of dying:

“… he’s always explained to me how I’m living with the cancer and it’s not going to kill me.” (Deidre; 60)

“… it’s just helpful knowing that it’s…a stage in my life which could go on for . many years.” (Deidre; 105)

“It’s probably an easier way of explaining it…because a lot of people think *‘Oh you’ve got cancer, you’re going to die.’* Shock, horror and all that, but it’s a useful way of erm explaining that you can go on for a long time.” (Deidre; 267)

Due to the chronic nature of her illness, Deidre is not able to distinguish a clear boundary between illness and wellness. Her accounts suggest however, that her anxieties about her health status are in some way contained by her consultant’s use of the concept “Living with cancer”.

1. Participant names have been changed for the purpose of anonymity in accordance with the signed confidentiality agreement. [↑](#endnote-ref-1)
